# Combined non-targeted and targeted metabolomics reveals the mechanism of delaying aging of Ginseng fibrous root

**DOI:** 10.3389/fphar.2024.1368776

**Published:** 2024-07-24

**Authors:** Xiang Yang, Xiang Yang, Bo Li, Jianyun Zhang, Zhuyun Yan

**Affiliations:** ^1^ State Key Laboratory of Characteristic Chinese Medicine Resources in Southwest China, School of Pharmacy, Chengdu University of Traditional Chinese Medicine, Chengdu, China; ^2^ Shijiazhuang Food and Drug Inspection Center, Shijiazhuang, China; ^3^ Beijing Apex Pharmaceutical R&D Co., Ltd., Beijing, China; ^4^ School of Pharmacy, Sichuan College of Traditional Chinese Medicine, Mianyang, China

**Keywords:** ginseng fibrous root, delaying aging, biochemical indicator, metabolomics, liquid chromatography-mass spectrometry

## Abstract

**Background:** The fibrous root of ginseng (GFR) is the dried thin branch root or whisker root of Ginseng (*Panax ginseng* C. A. Mey). It is known for its properties such as tonifying qi, producing body fluid, and quenching thirst. Clinically, it is used to treat conditions such as cough, hemoptysis, thirst, stomach deficiency, and vomiting. While GFR and Ginseng share similar metabolites, they differ in their metabolites ratios and efficacy. Furthermore, the specific role of GFR in protecting the body remains unclear.

**Methods:** We employed ultra-high performance liquid chromatography-triple quadrupole mass spectrometry to examine alterations in brain neurotransmitters and elucidate the impact of GFR on the central nervous system. Additionally, we analyzed the serum and brain metabolic profiles of rats using ultra-high performance liquid chromatography-quadrupole-orbitrap mass spectrometry to discern the effect and underlying mechanism of GFR in delaying aging in naturally aged rats.

**Results:** The findings of the serum biochemical indicators indicate that the intervention of GFR can enhance cardiovascular, oxidative stress, and energy metabolism related indicators in naturally aging rats. Research on brain neurotransmitters suggests that GFR can augment physiological functions such as learning and memory, while also inhibiting central nervous system excitation to a certain degree by maintaining the equilibrium of central neurotransmitters in aged individuals. Twenty-four abnormal metabolites in serum and seventeen abnormal metabolites in brain could be used as potential biomarkers and were involved in multiple metabolic pathways. Among them, in the brain metabolic pathways, alanine, aspartate and glutamate metabolism, arginine and proline metabolism, histidine metabolism, and tyrosine metabolism were closely related to central neurotransmitters. Butanoate metabolism improves energy supply for life activities in the aging body. Cysteine and methionine metabolism contributes to the production of glutathione and taurine and played an antioxidant role. In serum, the regulation of glycerophospholipid metabolism pathway and proline metabolism demonstrated the antioxidant capacity of GFR decoction.

**Conclution:** In summary, GFR plays a role in delaying aging by regulating central neurotransmitters, cardiovascular function, oxidative stress, energy metabolism, and other aspects of the aging body, which lays a foundation for the application of GFR.

## Introduction

Aging is a biological process which is affected by many complex factors such as heredity, environmental condition and life style (Declerck and Vanden, 2018). Due to the degenerative changes of various organs or viscera, the body’s natural aging leads to abnormal function and even diseases (Niccoli and Partridge, 2012). With the aging of the body, central activity, motor function, sensory function and so on will decline, accompanied by cardiovascular diseases, cancer, neurodegenerative diseases and other diseases, seriously affecting health and quality of life. By 2050, the global elderly population is expected to reach 1.6 billion, and the proportion will rise to 16% (Fan et al., 2009). Although aging is irreversible, it can be mitigated. Therefore, screening and development of drugs to delay aging, prevent and treat the age-related diseases is still one of the key topics of concern.

The fibrous root of ginseng (GFR) is the dried thin branch root or whisker root of Ginseng (*Panax ginseng* C. A. Mey), which was originally described in the “Bencao Fengyuan,” with tonifying qi, producing body fluid, quenching thirst and other effects. It is used in the clinic to treat cough, hemoptysis, thirst, stomach deficiency, and vomiting. Ginseng is commonly used as a traditional medicine and functional food for the prevention and treatment of a variety of diseases (Mancuso and Santangelo, 2017), such as oxidative damage (Park et al., 2021), immunomodulatory function and inflammation (Liu J. et al., 2019), the neurodegenerative disease and central fatigue related with central nervous system (Cheng et al., 2005; Jiang et al., 2017; Wang et al., 2021), cardiovascular diseases (Wang et al., 2021), cancer (Shin et al., 2000; Chang et al., 2003). GFR and ginseng have similar metabolites, but in different proportions, such as total saponins (G/GFR:4.4%/9.9%), PPD(Rb1+Rc + Rd)/PPT (Re + Rg1) (G/GFR:1.2/3.8), and Rb1/Rg1 (G/GFR:2.4/11.9), etc. (Yu et al., 2002). The sources of GFR are wide and the price is low, however, the effects of GFR on delaying aging and underlying mechanisms have rarely been reported.

Metabolomics utilizes analytical instruments with high separation efficiency, high sensitivity and low detection limits to focus on high-throughput identification and quantitative analysis of small molecule metabolites (≤1,500 Da) in living organisms (Beger et al., 2016). Through the dynamic changes of small molecule endogenous metabolites, the trend of physiological and pathological changes of the body (Barnes et al., 2016), the identification and quantification of metabolite biomarkers for disease pathogenesis (Johnson et al., 2016), diagnosis and prognosis are illustrated from the whole (Chen et al., 2021). Metabolomics provides new techniques for the screening and early diagnosis of complex diseases, as well as the regularity of disease progression and pharmacodynamic relationships.

In this study, we first performed untargeted metabolomics analysis of serum and brain tissue using liquid chromatography-quadrupole-Orbitrap MS (UHPLC-Q-Exactive Orbitrap MS) technology to screen for potential biomarkers related to aging. Then, the neurotransmitters in serum and brain of naturally aged rats were measured by liquid chromatography-triple quadrupole mass spectrometry (UPLC-Xevo TQ-XS) after administration of GFR decoction. Finally, the levels of aging-related biochemical indicators, such as those of the neuroendocrine system, cardiovascular system, oxidative stress, and energy metabolism were measured by enzyme-linked immunosorbent assay (ELISA) and microassay kits. The expected results are helpful for elucidatingthe possible role and mechanism of GFR in delaying aging related changes, and provide basic data for expanding the development and utilization of ginseng resources.

## Materials and methods

### Materials and reagents

Ginseng fibrous root was obtained from 6-year-old Ginsengs under Forest purchased from Meijiayun Chinese Medicinal Materials Co., Ltd. (Anguo, China). Six-year-old Ginsengs under Forest were identified by Professor Yan Zhuyun.Valine-13C5-15 N and leucine-13C6 were purchased from Sigma-Aldrich (Shanghai, China). Phenylalanine-d5,3-chloro-D-phenylalanine, octanoic-d15 acid, decanoic-d19 acid, octadecanoic-d35 acid, tetradecanoic-d27 acid, hexadecanoyl-L-carnitine-d3 and decanoyl-L-carnitine-d3 were obtained from C/D/N Isotopes (Pointe-Claire, Quebec, Canada). The reference compounds for identification were mainly purchased from Sigma-Aldrich (Shanghai, China), Santa Cruz Biotechnology (Shanghai, China), Toronto Research Chemicals (Toronto, Ontario, Canada), and Aladdin Bio-Chem Technology (Shanghai, China). LC-MS grade acetonitrile, methanol, and formic acid were the products of Fisher Scientific. Other materials were obtained from Shanghai Anpel Laboratory Technologies (Shanghai, China).

### Preparation of GFR decoction

Fifty grams of GFR granules were decocted with 400 mL, 300 mL and 200 mL of water 3 times for 2 h, 1 h, and 1 h, respectively. Then, the decoction was filtered and concentrated to 50.0 mL.

### Experimental animals

All animal experiments were performed in accordance with the principles of laboratory animals and under the guidance of the Animal Ethics Committee of Chengdu University of Traditional Chinese Medicine (license number: SYXK (Chuan) 2020–124). Healthy male Sprague-Dawley rats, aged 6 weeks, 8 rats (180–220 g), and sixteen animals (420–450 g), aged 18 months, were purchased from SPF (Beijing) Biotechnology Co., Ltd. (Animal Certificate No.: SCXK [Beijing] 2019–0010). Rats were kept at 24°C ± 1°C and 45% ± 15% relative humidity under a 12/12 h light-dark cycle. The rat were fed a standard diet and had free access to water.

### Animal handling and sample collection

After 2 weeks of adaptation, 6-week-old rats served as the control group (C). Sixteen 18-month-old rats were randomly divided into 2 groups (n = 8): the model group (M) and the GFR group (GFR). The GFR group received GFR decoction (9.5 mL/kg, ig) once, and the control group and the model group were given the same volume of pure water. All rats were anesthetized by intraperitoneal injection of 1% sodium pentobarbital (0.15 mL/100 g) at 2 h after administration and sacrificed, and abdominal aortic blood and brain tissues were collected immediately. Blood samples were centrifuged at 4,000 rpm for 15 min and serum samples were collected. Brain tissue was placed in normal saline (NS) to remove residual blood. Serum and brain samples were stored in −80°C in an ultra-low temperature refrigerator.

### Determination of biochemical indicators

The preserved serum samples were removed, thawed at room temperature and tested using commercial test kits according to the manufacturer’s protocol. Creatine kinase (CK), malondialdehyde (MDA), succinate dehydrogenase (SDH), lactate dehydrogenase (LDH), natriuretic peptide/brain natriuretic peptide (BNP), rat adrenocorticotropic hormone (ACTH), cortisol (CORT), aldosterone (ALD), epinephrine (E), norepinephrine (NE), angiotensin II (Ang II) and superoxide dismutase (SOD) were provided by Shanghai Enzyme Linked Biotechnology Co., Ltd. (Shanghai, China).

### Instruments and conditions

#### Untargeted metabolomics

The samples were analyzed by a ThermoFisher Ultimate 3000 UHPLC system and a ThermoFisher Q Exactive™ Hybrid Quadrupole-Orbitrap™ Mass Spectrometry (QE). Samples were separated by chromatography using an ACQUITY UPLC BEH C18 column (2.1 mm × 100 mm, 1.7 μm, Waters). The mobile phases consisted of (A) water with 0.1% formate and (B) acetonitrile with 0.1% formate. Linear gradient elution was performed with the following program: 0–1 min, 1% B; 3 min, 15% B; 5 min, 60% B; 7.5–12 min, 100% B; 12.1–14 min, 1% B. The flow rate was 0.4 mL/min. A heated electrospray ion source (HESI) was used to ionize the metabolites in positive and negative ion modes, and the main parameters were set as follows: the spray voltage was set to 3,200 V. The capillary and probe heater temperatures were 320°C and 350°C, respectively. The sheath gas flow rate was 40 (Arb, arbitrary unit), and the Aux gas flow rate was 10 (Arb) for positive mode. The S-Lens RF Level was 50 (Arb). The full scan was operated at a high-resolution of 35,000 FWHM (*m/z* = 200) in the range of 70–1050 *m/z* with AGC Target setting at 3 × 10^6^. Simultaneously, the fragment ion information of the top 10 precursors in each scan was acquired by Data-dependant acquisition (DDA) with HCD energy at 15, 30, and 45 eV, a mass resolution of 17,500 FWHM, and AGC Target of 1 × 10^6^.

#### Quantitative determination of brain neurotransmitters

UHPLC-MS/MS analysis was performed on a Waters ACQUITY UPLC H-Class UHPLC system coupled to a Xevo TQ-XS Triple Quadrupole mass spectrometer (Milford, MA, United States). Samples were injected into a Waters HSS T3 column (100 mm × 2.1 mm, 1.7 μm) at a flow rate of 0.4 mL/min. The mobile phase consisted of (A) water with 0.1% formate and (B) acetonitrile with 0.1% formate. A linear gradient was used for chromatographic separation: 1%–100% B. The eluted analysts were ionized in an electrospray ionization source in positive mode (ESI+) and negative mode (ESI-). The source temperature was 150°C. The desolvation gas temperature was 450°C. The desolvation gas flow was 900 L/h. The cone gas flow was 50 L/h, and the capillary voltage was 1,000 V. Multiple reaction monitoring (MRM) was used to acquire data in the optimized MRM transition (precursor - > product), cone voltage (CV), and collision energy (CE), as shown in Table 1. Masslyxn 4.2 software was used to control instruments and acquire data.

**TABLE 1:** Optimized multiple reaction monitoring (MRM) parameters, Cone voltage (CV), and collision energy (CE) for neurotransmitters and internal standard (IS).

No.	Analyte	Name	Precursor ion (*m/z*)	Product ion (*m/z*)	Cone voltage (V)	Collision energy (eV)
1	Trp	Tryptophan	205.143	146.015	20	20
2	5-HTrp	5-Hydroxytryptophan	221.100	204.100	25	20
3	N-AS	N-Acetylserotonin	219.032	117.000	20	40
4	5-HIAA	5-Hydroxyindoleacetic acid	190.096	146.049	20	30
5	Tra	Tryptamine	161.100	144.100	15	20
6	Trpo	Tryptophol	162.100	144.100	25	20
7	Kyn	Kynurenine	207.050	190.040	15	10
8	Kya	Kynurenic acid	188.096	144.042	20	15
9	3-Haphe	3-Hydroxyanthranilic acid	152.032	108.008	20	15
10	5-HT	5-Hydroxytryptamine	177.100	160.100	15	20
11	Orn	Ornithine	133.030	69.998	20	20
12	Gln	Glutamine	147.054	55.954	20	40
13	GABA	Gamma-Aminobutyric acid	103.968	68.716	10	15
14	SA	Succinic acid	116.930	73.028	20	10
15	Phe	L-Phenylalanine	166.094	102.952	20	30
16	Pht	Phenylethylamine	122.032	105.033	20	15
17	Tyr	L-Tryosine	182.094	91.037	20	25
18	Trya	Tyramine	137.968	76.884	15	20
19	L-Dopa	L-Dopa	198.100	152.050	30	20
20	DOPN	Dopamine	154.000	109.000	30	15
21	DHPA	3,4-Dihydroxyphenylacetic acid	167.032	123.000	10	10
22	VMA	Vanillylmandelic acid	197.032	137.000	15	30
23	Norp	Norepinephrine	152.100	94.700	15	20
24	Asp	Aspartic acid	132.000	88.000	15	15
25	Ind-3-C	Indole-3-carboxaldehyde	144.000	115.000	20	25
26	QA	Quinolinic acid	165.960	121.900	20	10
27	IAA	Indoleacetic acid	174.100	130.000	15	10
28	Ind-3-L	3-(3-indolyl) lactic acid	206.200	130.000	15	30
29	Adr	Adrenaline	184.000	166.000	15	10
30	Mel	Melatonine	233.100	174.100	25	15
31	HA	Histamine	112.000	95.000	20	10
32	5-MT	5-Methoxytryptamine	191.143	174.000	10	10
33	NFK	N-Formylkynurenine	237.100	136.000	20	20
34	XTA	Xanthurenic acid	204.030	116.057	15	30
35	ACh	Acetylcholine	146.000	87.000	25	15
36	Glu	Glutamic acid	148.000	84.000	10	20
37	IS	13C11-Trp	216.000	155.000	10	20

#### Preparation of biological samples

Sample preparation for non-targeted metabolomics analysis: Serum samples and 200 μL of methanol (containing 250 ng/mL internal standards) were added to 50 μL of each serum sample and vortexed. Then, the mixture was allowed to stand at −40°C for 30 min and 4°C for 10 min and centrifuged at 15,000 *g* and 4°C for 15 min. The supernatant was evaporated to dryness and reconstituted in 50 μL of 30% methanol prior to UHPLC-HRMS/MS analysis. A quality control (QC) sample was obtained by isometrically pooling all the prepared samples.

Brain tissue samples were homogenized in liquid nitrogen in a mortar. A total of 50 mg of the brain was added to a 20-fold volume of MTBE/methanol/water solvent (μL/mg, v/v/v = 10:32:8) containing 200 ng/mL of internal standard. Following centrifugation at 15,000 *g* and 4°C for 15 min, 300 μL of supernatant was evaporated to dryness and reconstituted in 60 μL of 30% methanol prior to UHPLC-HRMS/MS analysis. A quality control (QC) sample was obtained by isometrically pooling all the prepared samples.

Preparation of the sample for the targeted metabolomics analysis of brain neurotransmitters: 50 mg of the brain tissue was added to 300 μL of 80% methanol solvent (μL/mg) containing 100 ng/mL tryptophan-13C11 as an internal standard. Following centrifugation at 15,000 *g* and 4°C for 15 min, all supernatant was collected. The extraction was repeated with 200 μL of 80% methanol, and the supernatants from the two extractions were combined. Then, 300 μL chloroform was added. After 30 min at −4°C, the mixture was centrifuged 15,000 *g* for 15 min at 4°C. An 80% upper phase (400 μL) was evaporated to dryness and reconstituted in 80 μL of 50% methanol prior to UHPLC-MS/MS analysis. The quality control (QC) sample was obtained by isometrically pooling all the prepared samples.

## Detection of main saponins in ginseng fibrous root decoction

In this study, the water decoction of the Fibrous root of Ginseng (*P. ginseng* C. A. Mey) was used as the object. According to the method of Ginseng content determination in the first part of *Chinese Pharmacopoeia*, the concentrated water decoction was extracted by adding 50 mL water saturated n-butanol, and the n-butanol layer was taken and dried in the evaporation dish. The residue was dissolved with methanol and filtered, and then put into shipping containers. In waters 2965 chromatographic system (2489 Detector, waters, United States; ELSD 6000, Alltech, United States), Gemini 5μ-C18 (250 × 4.6 mm, 5 μm) was used, with the gradient elution procedure and UV detector parameters according to the Ginseng content determination item of Chinese Pharmacopoeia. For ELSD, the carrier gas flow rate was set at 2.9 L•min^−1^, the drift tube temperature was set at 107°C and the gain was set at 1. In LC-MS (TSQ Quantis, Thermo Fisher Scientific, United States), Waters HSS T3-C18 (2.1 × 100 mm, 1.8 μm) column was used, with a gradient elution of A: acetonitrile; and B: water with 0.1% HCOOH (v/v); A: B 0 min 90:10 (v/v), in 4th min 2% A, in 6th min 2% A. The flow rate was 0.3 mL/min, column block temperature was 30°C. MS conditions: sheath gas 40 L/min, aux gas 10 L/min, Vaporizer temperature 350°C, ion spray voltage −3,500 V, scan mode m/z 200–1250 were identified by comparison of retention times, UV, ELSD and MS profile with standards. The quantification was carried out by using calibration curves constructed based on measurements of corresponding standards.

### Data processing and statistical analysis

UPLC-QE-MS/MS data were converted into mzXML format by ProteoWizard software, and then peak identification, peak alignment, retention time correction, and peak annotation were performed by the XCMS and CAMERA software packages on the R software platform for untargeted metabolomics analysis. Volcano plot analysis was used to screen differentially abundant metabolites. The selection criteria were a unidimensional *p*-value of the statistical analysis (*p* < 0.05, namely, -log10*p*>1.30) in combination with multiple changes in FC (FC > 1.2, namely, |log2FC|>0.26). Structure identification was performed using the Human Metabolome database (https://www.hmdb.ca/), mzCloud (mzCloud–Advanced Mass Spectral Database), MoNA (https://mona.fiehnlab.ucdavis.edu/), and Kyoto Encyclopedia of Genes and Genomes databases (http://www.kegg.jp/). The online software MetaboAnalyst (version 5.0, http://www.metaboanalyst.ca/) was used for pathway analysis of differential metabolites. Data are presented as mean ± standard error of mean (SEM). Statistical analyses were performed using IBM SPSS Statistics 25 (SPSS Inc., Chicago, IL, United States). ^*^ *p* < 0.05 and ^#^ *p* < 0.05 indicates a significant difference, ^**^ *p* < 0.01 and ^##^ *p* < 0.01 indicates a highly significant difference, and ns indicates no significant difference.

## Results

### Evaluation of main saponins in ginseng fibrous root decoction

HPLC and MS techniques were used to analyze the pharmacopoeia indicator ginsenosides in ginseng fibrous root decoction. Three detectors confirmed the presence of ginsenosides Rb1, Rg1 and Re (Supplementary Figure S1). The metabolites were identified by comparing the UV spectrum with the evaporative light scattering spectrum and the retention time of the corresponding standard. Furthermore, we quantified these substances in the extract using LC-MS. The concentrations of Rg1, Re, and Rb1 in GFR were 0.18%, 0.40%, and 0.90% respectively, thereby satisfying the ginsenoside content standards for Radix Ginseng as outlined in the Chinese Pharmacopoeia.

### Effects of GFR on serum biochemical indicators

The results of ageing-related indicators in rat serum (Table 2) revealed that when compared to the control group of 6-month-old rats, there was a significant increase in 10 biochemical indicators and a decrease in 2 biochemical indicators in the model group of 18-month-old rats. However, all these indicators were significantly reversed in the GFR group. The serum BNP level of 18-month-old rats (model group) was significantly higher than that of 6-month-old rats (control group) (*p* < 0.01), and the BNP level of GFR group was significantly lower than that of model group (*p* < 0.01). The serum levels of ACTH, CORT, NE, EPI, AngⅡ, and ALD in the model group were higher than those in the control group (*p* < 0.01), and the other indicators except CORT in the GFR group were significantly improved. The activities of LDH and CK in serum of model group were significantly higher than those of control group (*p* < 0.01), but the activities of SDH were opposite. The above indicators in GFR group were significantly reduced (*p* < 0.01), indicating that the energy metabolism of aged rats was improved after treatment with GFR. The serum MDA level of the model group was significantly higher than that of the control group (*p* < 0.01), and the SOD activity was significantly lower than that of the control group (*p* < 0.01). These two biochemical indicators in GFR group were significantly improved. These results suggest that GFR can improve the biochemical indexes related to aging in 18-month-old rats by improving the cardiac function, regulating the sympathetic adrenal medulla axis (SAM), (sympathetic) renin-angiotensin-aldosterone system, energy metabolism and antioxidant activity after the intervention of GFR in aged rats.

**TABLE 2:** Effect of GFR on the markers of aged rats (n = 8).

Group	BNP	ACTH	EPI	NE	AngⅡ	ALD	SOD	MDA	CORT	CK	LDH	SDH
	(pg/mL)	(pg/mL)	(ng/mL)	(ng/mL)	(pg/mL)	(pg/mL)	(ng/mL)	(nmol/mL)	(ng/mL)	(nmoL/h/mL)	(nmol/min/mL)	(nmol/min/mL)
C	335.85 ± 35.34	38.23 ± 2.47	7.28 ± 0.57	3.35 ± 0.18	699.37 ± 36.92	398.27 ± 10.54	7.14 ± 0.46	8.57 ± 1.89	73.28 ± 3.76	1.77 ± 0.24	8.59 ± 2.29	394.25 ± 19.06
M	731.75 ± 47.18^**^	77.81 ± 2.38^**^	15.92 ± 0.70^**^	7.18 ± 0.24^**^	1234.36 ± 44.15^**^	654.17 ± 31.00^**^	5.08 ± 0.35^**^	41.31 ± 2.10^**^	101.49 ± 6.60^**^	4.90 ± 0.47^**^	37.61 ± 2.87^**^	196.44 ± 18.48^**^
GFR	408.50 ± 35.50^##^	55.31 ± 2.15^##^	7.94 ± 0.18^##^	4.10 ± 0.14^##^	796.25 ± 40.07^##^	431.50 ± 9.62^##^	6.76 ± 0.34^##^	19.11 ± 2.52^##^	99.45 ± 4.18^ns^	2.97 ± 0.27^##^	15.96 ± 1.79^##^	343.78 ± 22.03^##^

Significant differences were determined by one-way analysis of variance. The data are expressed as the mean ± SEM (n = 8). *p < 0.05, **p < 0.01 relative to the C group; #*p* < 0.05, ##*p* < 0.01 relative to the M group, ns indicates no significant difference. BNP: brain natriuretic peptide; ATCH, Adrenocorticotrophic hormone; EPI, epinephrine; NE, noradrenaline; AngⅡ, Angiotensin II; ALD, aldosterone; SOD, superoxide dismutase; MDA, malondialdehyde; CORT, cortisol; CK, creatine kinase; LDH, lactate dehydrogenase; SDH, succinate dehydrogenase.

### Effects of GFR on neurotransmitters in brain tissue

**FIGURE 1:**
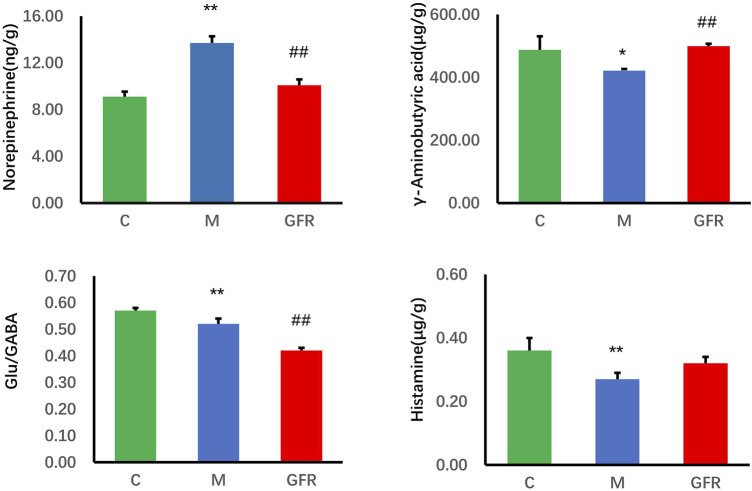
Content of neurotransmitter in brain tissue of each group. Data were expressed as the mean ± SEM (n = 8). ^*^ *p <* 0.05, ^**^ *p <* 0.01 relative to the C group; ^#^ *p <* 0.05, ^##^ *p <* 0.01 relative to the M group. C: normal control group; M: model control group; GFR: model administration group.

The results of targeted metabolomics analysis of neurotransmitters in rat brain tissues (Table 3) showed that 8 neurotransmitters, related metabolites and their related ratios in the model group were significantly different from those in the control group. Among them, the levels of Glu, 5-HT, GABA, DA and HA were significantly decreased (*p* < 0.01 or *p* < 0.05). The levels of Trp, Phe and NE were significantly increased (*p* < 0.01 or *p* < 0.05). The ratios of Glu/GABA, 5-HT/NE, 5-HT/DA, and 5-HT/5-HIAA were significantly decreased (*p* < 0.01 or *p* < 0.05). In rat brain tissue of GFR NE and GABA rebounded significantly (*p* < 0.01), HA showed some degree of rebound, and Trp, Phe and Glu/GABA significantly exacerbated the changes observed in the model group indicators, while others remained relatively unchanged (Figure 1). In the pathways of Phenylalanine, tyrosine, and tryptophan biosynthesis, phenylalanine serves as the precursor for the synthesis of tyrosine and tryptophan. Tryptophan can be further metabolized into 5-hydroxytryptamine (5-HT). However, as phenylalanine levels continuously increased, tyrosine levels did not change significantly, tryptophan levels significantly increased, but 5-HT levels continuously decreased. This suggests that GFR did not improve this indicator and metabolic pathway. These results suggest that GFR can regulate the level of certain neurotransmitters in the brain tissue of aged rats after intervention.

**TABLE 3:** Effect of GFR on the brain neurotransmitter indicators of aged rats (n = 8).

Group	Glu (μg/g)	Trp (μg/g)	5-HT (μg/g)	GABA (μg/g)	Phe(μg/g)	DA (μg/g)	HA (μg/g)	NE (ng/g)	Glu/GABA	5-HT/NE	5-HT/5-HIAA	5-HT/Trp
C	239.60 ± 5.87	78.49 ± 2.70	2.38 ± 0.41	487.74 ± 43.29	191.91 ± 6.79	2.12 ± 0.37	0.36 ± 0.04	9.10 ± 0.44	0.57 ± 0.01	0.26 ± 0.04	0.70 ± 0.11	0.03 ± 0.01
M	218.40 ± 8.80*	87.47 ± 2.37*	1.32 ± 0.22*	421.99 ± 4.94*	214.32 ± 4.62^*^	1.38 ± 0.17^*^	0.27 ± 0.02^**^	13.71 ± 0.56^**^	0.52 ± 0.02^*^	0.10 ± 0.02**	0.41 ± 0.06^*^	0.02 ± 0.00^*^
GFR	210.09 ± 5.81^ns^	121.31 ± 1.86^##^	1.13 ± 0.11^ns^	499.57 ± 7.14^##^	323.00 ± 7.80^##^	1.38 ± 0.18 ^ns^	0.32 ± 0.02 ^ns^	10.09 ± 0.49^##^	0.42 ± 0.01^##^	0.11 ± 0.01 ^ns^	0.35 ± 0.04 ^ns^	0.01 ± 0.00 ^ns^

Significant differences were determined by one-way analysis of variance. Data were expressed as the mean ± SEM (n = 8). ^*^ *p <* 0.05, ^**^ *p <* 0.01 relative to the C group; ^#^ *p <* 0.05, ^##^ *p <* 0.01 relative to the M group. Glu, Glutamic acid; Trp, Tryptophan; 5-HT: 5-Hydroxytryptamine; GABA, Gamma-Aminobutyric acid; Phe, L-Phenylalanine; DA, dopamine; HA, histamine; NE, norepinephrine; 5-HIAA, 5-Hydroxyindoleacetic acid.

### Effects of GFR on biomarkers and their metabolic pathways

#### Multivariate data analysis of serum and brain samples

**FIGURE 2:**
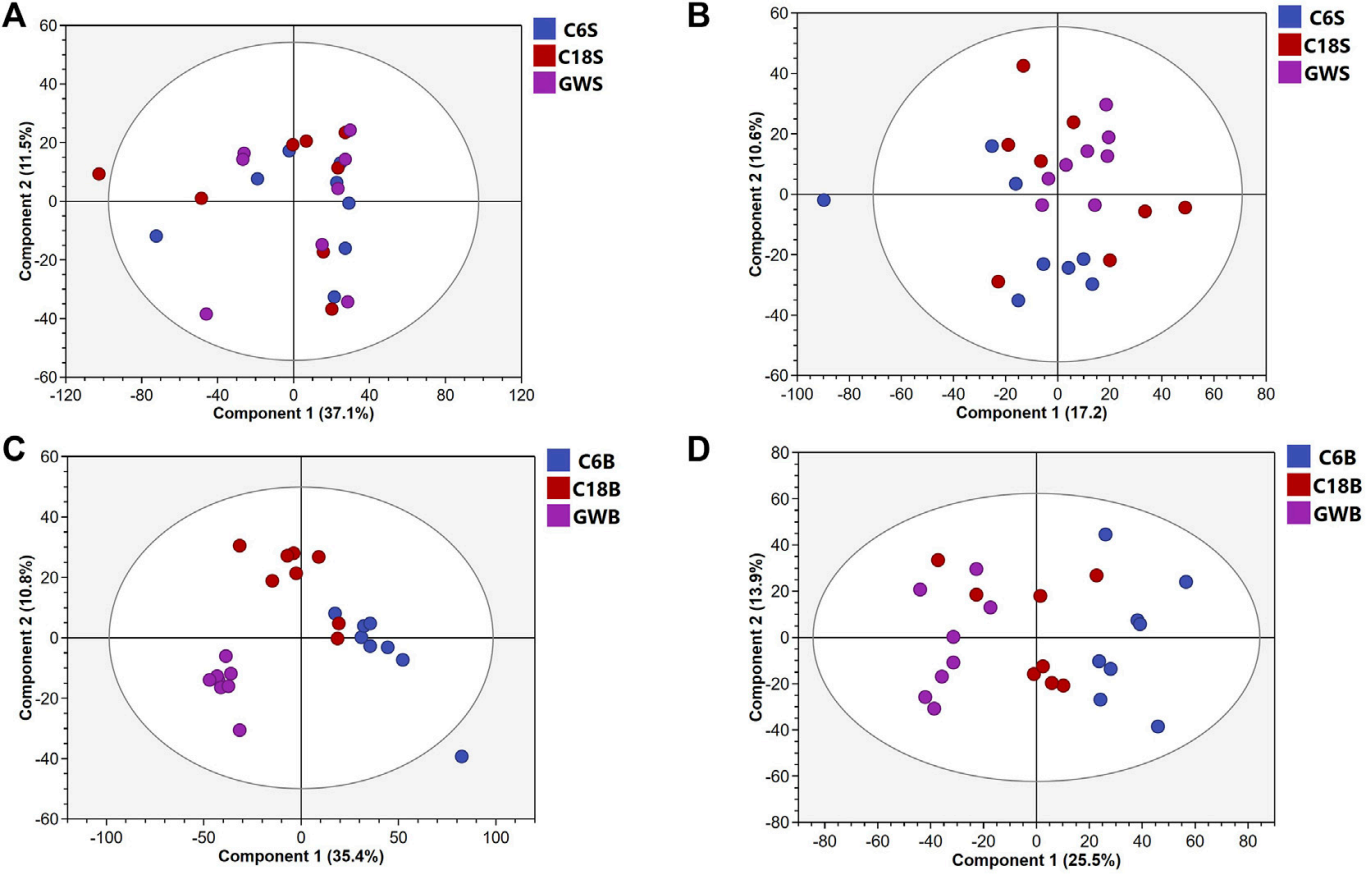
PCA score plots of serum samples from C, M and GFR in positive **(A)** and negative **(B)** ESI modes (n = 8); PCA score plots of brain samples from C, M and GFR in positive **(C)** and negative **(D)** ESI modes (n = 8). C6S and C6B: normal control group (C); C18S and C18B: model control group (M); GWS and GWB: model administration group (GFR).

**FIGURE 3:**
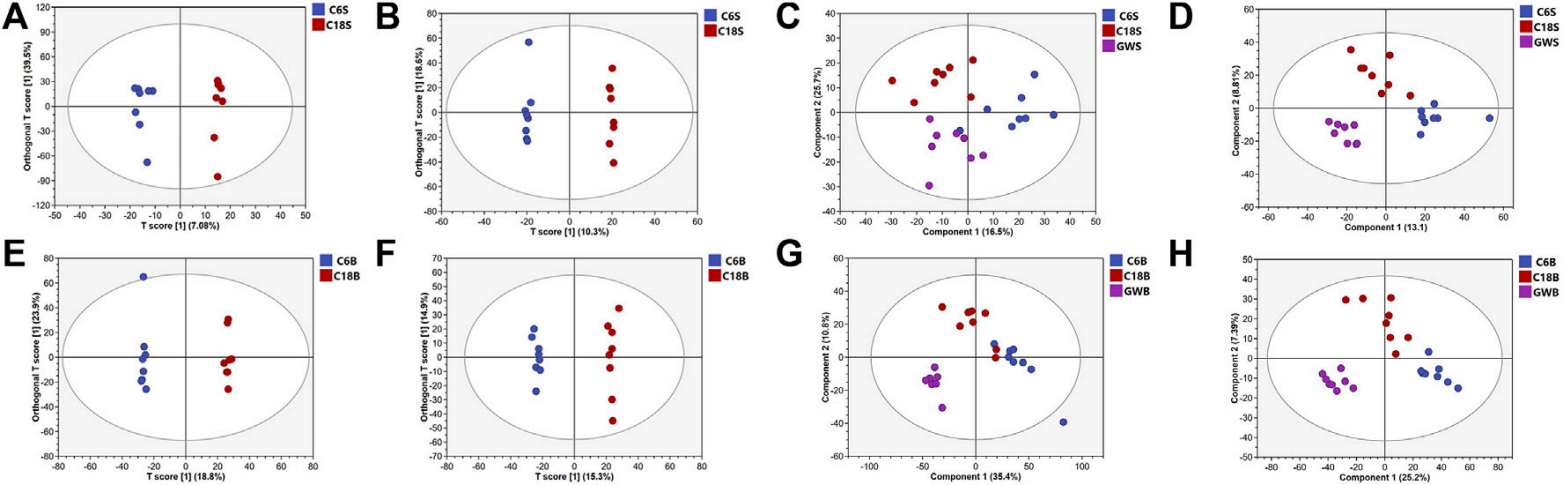
OPLS-DA and PLS-DA score plots of serum samples and brain samples. OPLS-DA score plots of serum samples from C and M in positive **(A)** and negative **(B)** ESI modes (n = 8); PLS-DA score plots of serum samples from C and M in positive **(C)** and negative **(D)** ESI modes (n = 8); OPLS-DA score plots of brain samples from C, M and GFR in positive **(E)** and negative **(F)** ESI modes (n = 8). PLS-DA score plots of brain samples from C, M and GFR in positive **(G)** and negative **(H)** ESI modes (n = 8); C6S and C6B: normal control group (C); C18S and C18B: model control group(M); GWS and GWB: model administration group (GFR).

UHPLC-QE-MS/MS metabolomics analysis was performed on serum and brain samples of rats with positive and negative ion modes, and the changes of GFR intervention in aged rats were explored from the metabolome level as shown in Figure 2. PLS-DA models of endogenous metabolites in serum and brain tissue (Figure 3) showed that the C, M, and GFR groups separated from each other in positive and negative ion modes, indicating significant differences in endogenous metabolites between them. Meanwhile, the model quality was evaluated by using R^2^Y and Q^2^ values, both of which are greater than 0.5, indicating that the model has good adaptability and predictability.

#### Identification of potential endogenous metabolites of serum and brain samples

**FIGURE 4:**
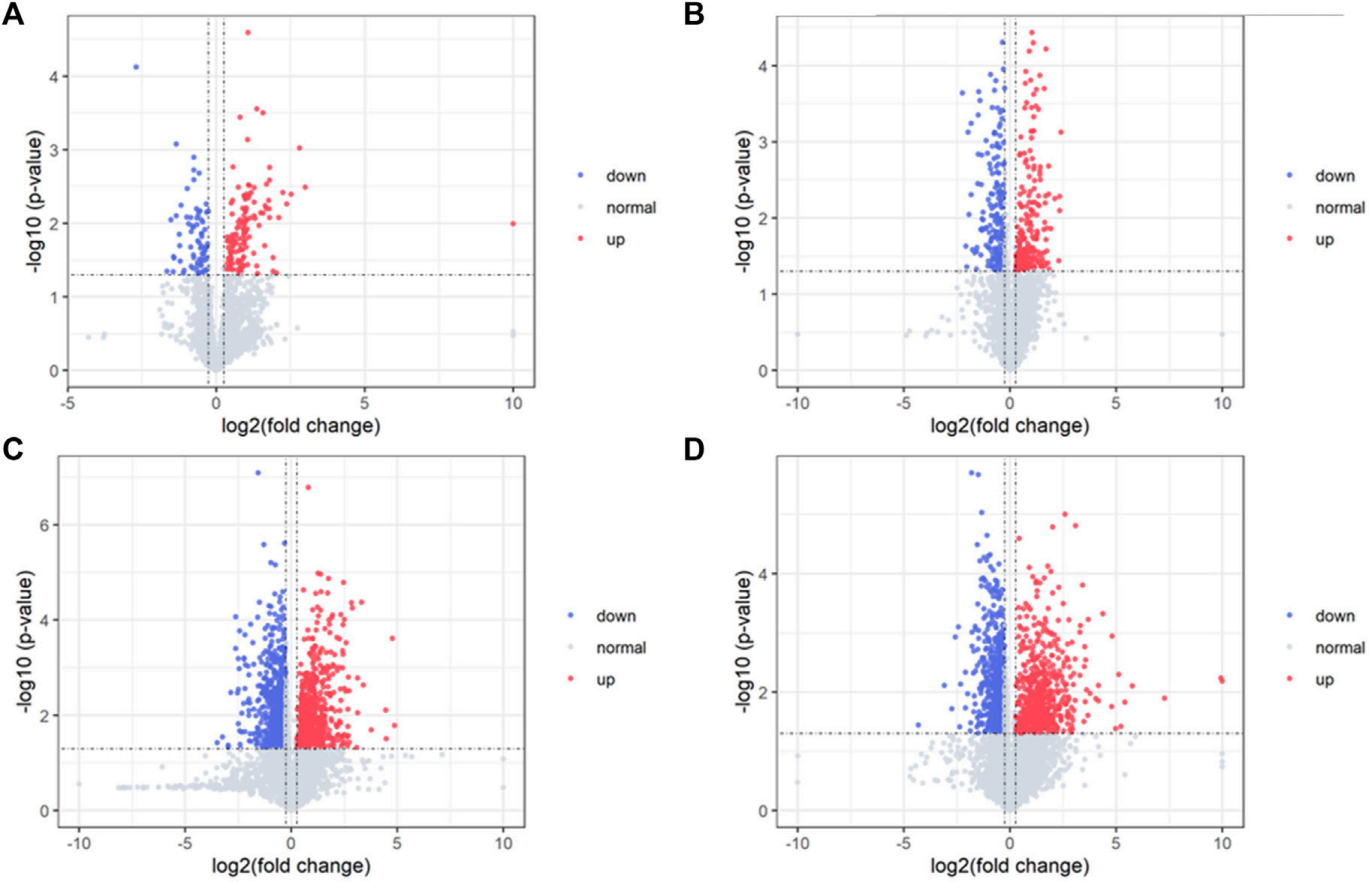
Volcano plot of serum samples and brain samples. Volcano plot of serum samples from C and M in positive **(A)** and negative **(B)** ESI modes (n = 8). Volcano plot of brain samples from C and M in positive **(C)** and negative **(D)** ESI modes (n = 8).

**FIGURE 5:**
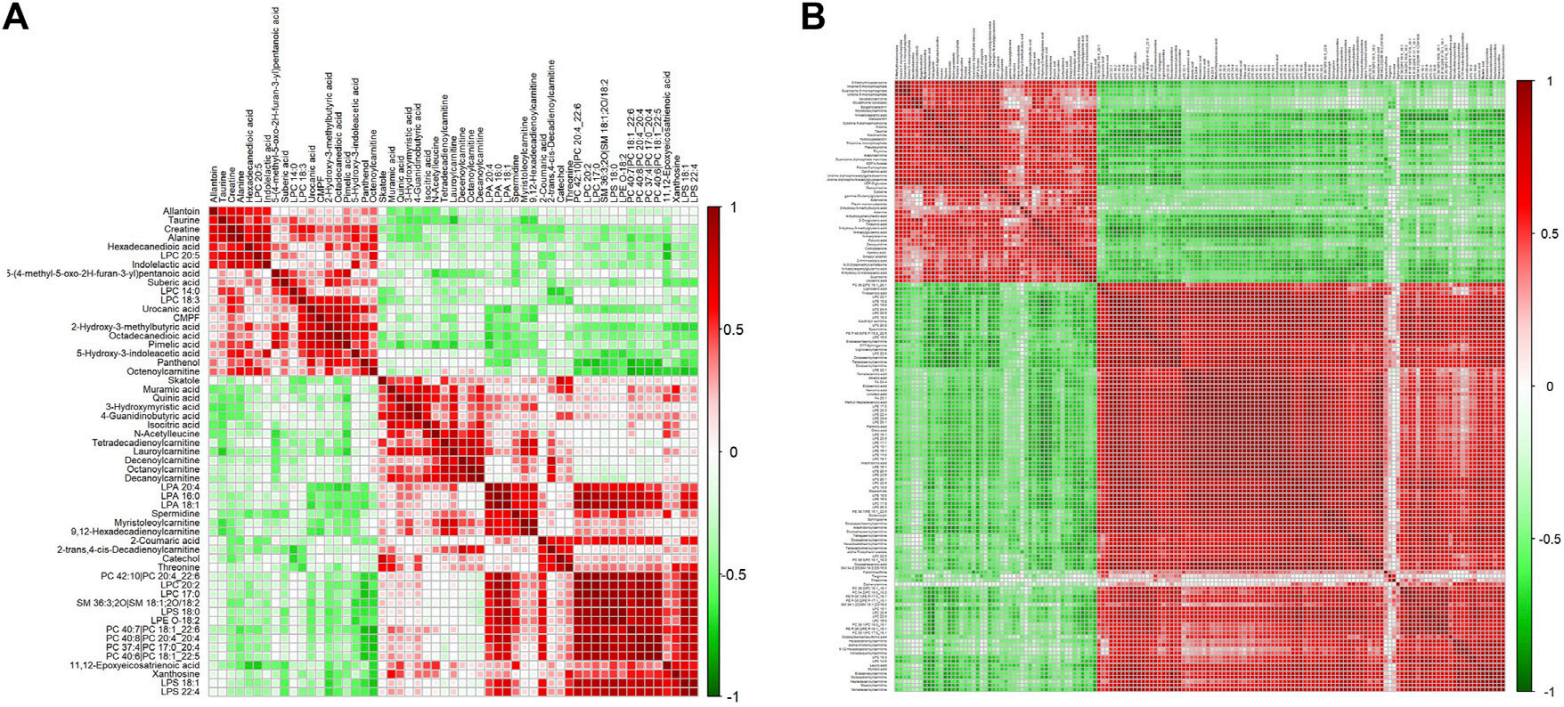
Correlation analysis of metabolites associated with GFR in M rats. Pearson correlation matrix analysis of serum **(A)** and brain **(B)** metabolites.

**FIGURE 6:**
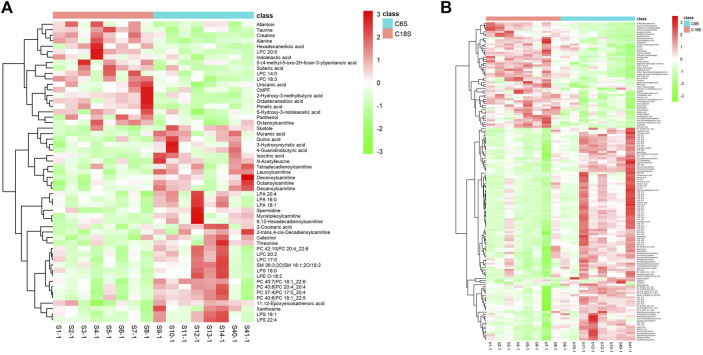
Differential analysis of metabolites associated with GFR efficacy in M rats. Cluster heat maps of serum **(A)** and brain **(B)** metabolites in each group.C6S and C6B: normal control group (C); C18S and C18B: model control group(M).

**FIGURE 7:**
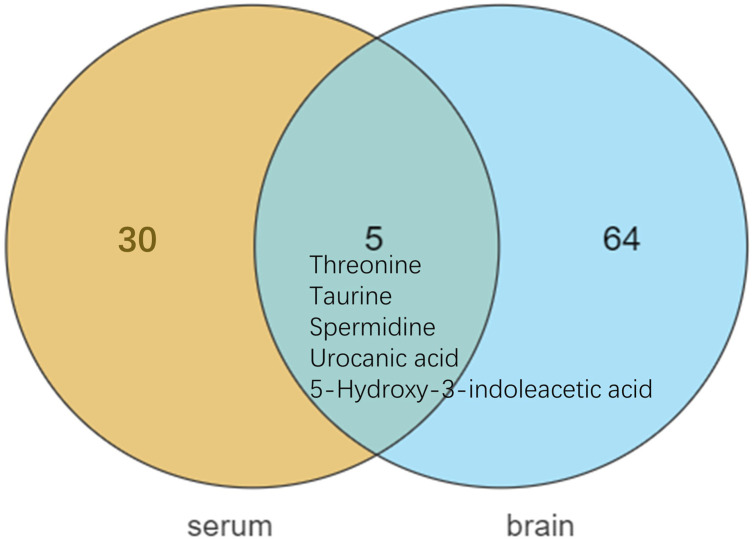
Metabolite differences between serum and brain samples.

Differential metabolites in serum and brain samples between groups C and M associated with aging were screened by volcano plots of variables (Figure 4). To use multiple alterations with the unidimensional statistical method of *p* values., the levels were set to *p* < 0.05 (-log10*p*>1.30) and FC > 1.2 (|log2FC|>0.26). There were 55 different endogenous metabolites in the serum samples and 151 different endogenous metabolites in the brain samples, of which 35 (Supplementary Table S1) and 69 (Supplementary Table S2) were identified in the HMBD and KEGG databases, respectively. After the administration of GFR, 24 and 17 metabolites, respectively, received varying degrees of callback. The results of Pearson correlation analysis of these substances are shown in Figure 5. As shown in Figure 5A, Phosphatidylcholine (PC 40:6|PC 18:1_22:5, PC 40:7|PC 18:1_22:6, PC 40:8|PC 20:4_20:4), lysophosphatidylcholine (LPC 17:0, LPC20:2), lysophosphatidic acid (LPA18:1) and spermine presented positive correlation. Figure 5B showed a positive correlation between 5′-Methyl-thioadenosine and taurine. The cluster analysis results of metabolites are shown in Figure 6. A Venn diagram was constructed for the analysis of endogenous metabolites in serum and brain samples (Figure 7), threonine, taurine, spermidine, urocanic acid, and 5-hydroxy-3-indoleacetic acid were detected in both the serum and brain samples.

#### Metabolic pathway analysis of potential endogenous metabolites in serum

**FIGURE 8:**
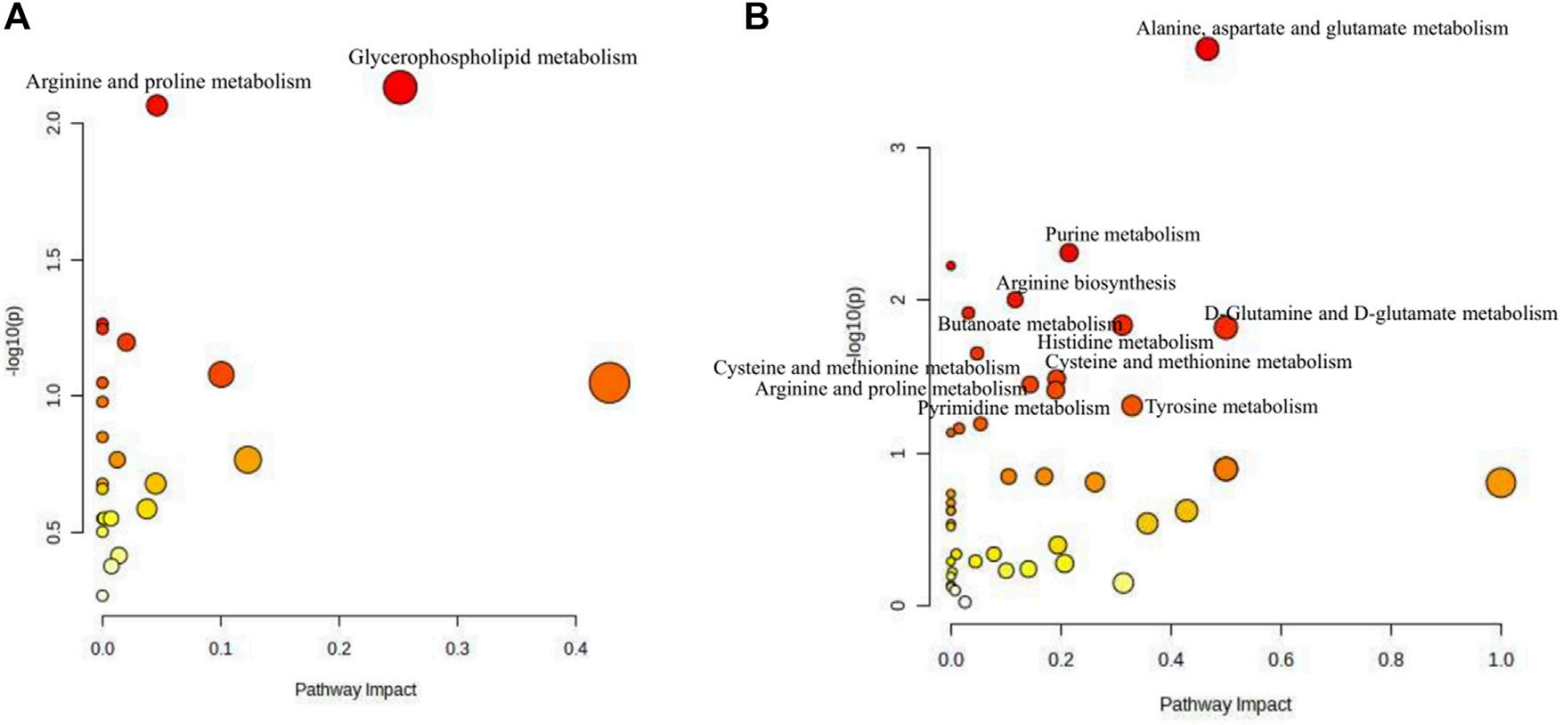
Pathway analysis of the aging-related metabolites. **(A)** Pathway analysis of serum. **(B)** Pathway analysis of brain.

**FIGURE 9:**
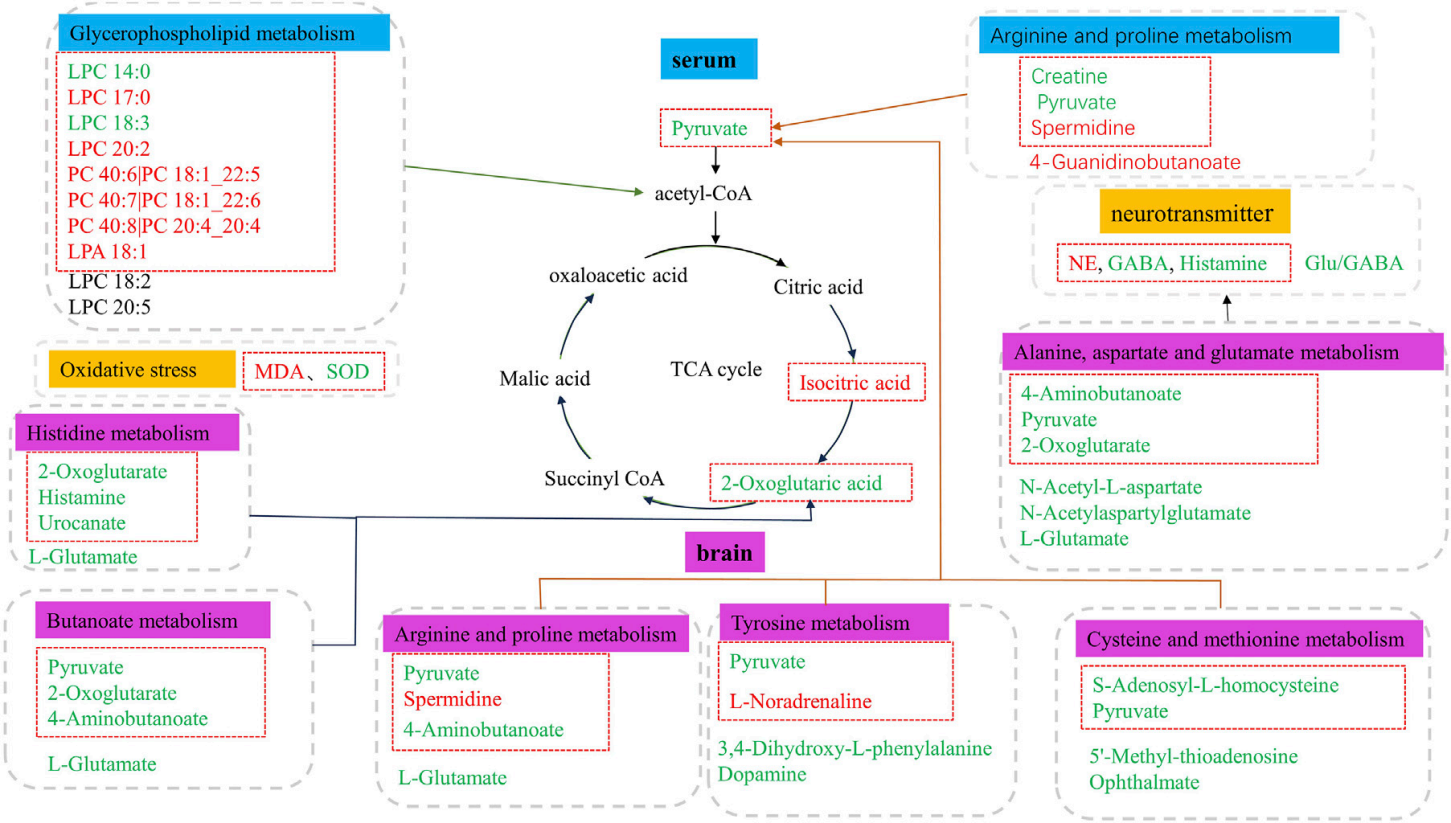
Metabolic disorder in aged model rats. The metabolites marked in red are potentially differential endogenous metabolites with elevated levels compared to Group C, and those with decreased levels compared to the Group C are in green. The differential metabolites in the red box indicate the callback under GFR.

Based on the differential metabolites identified in serum, the main biological metabolic pathway analysis was performed using the MetaboAnalyst 5.0 online database yielding 23 metabolic pathways. Of these, two metabolic pathways were pinpointed: Glycerophospholipid metabolism and Arginine and proline metabolism (*p* > 0.05, impact>0), and the results are shown in Figure 8A. The 24 metabolites and biochemical indicators of GFR callback were mainly involved in arginine and proline metabolism and glycerophospholipid metabolism, as detailed in Figure 9.

#### Metabolic pathway analysis of potential endogenous metabolites in brain

Based on the identified differential metabolites in brain, the main biological metabolic pathway analysis was performed using the MetaboAnalyst 5.0 online database, yielding 45 metabolic pathways. Of these, 11 metabolic pathways exhibited (*p* > 0.05, impact>0). These include Alanine, aspartate and glutamate metabolism; Purine metabolism; Arginine biosynthesis; Butanoate metabolism; Histidine metabolism; D-Glutamine and D-glutamate metabolism; Cysteine and methionine metabolism; Amino sugar and nucleotide sugar metabolism; Arginine and proline metabolism; Pyrimidine metabolism; and Tyrosine metabolism. The findings are depicted in Figure 8B. The 17 metabolites and neurotransmitters of GFR reduction were mainly involved in 6 metabolic pathways. They were alanine, aspartate, and glutamate metabolism, cysteine and methionine metabolism, arginine and proline metabolism, histidine metabolism, and tyrosine metabolism, and butanoate metabolism. Notably, the first five pathways pertain to amino acid metabolism, while the last one is associated with carbohydrate metabolism, as illustrated in Figure 9.

## Discussion

Aging is a complex natural phenomenon. The physiological changes in the aging process of human body are mainly reflected in the loss of body tissues and cells and constituent substances, the decrease of body metabolic rate, and the decline of body and organ function (Nishiura et al., 2022). The present study showed that the serum levels of ACTH, CORT, NE, EPI, AngⅡ, and ALD in 18-month-old rats were significantly higher than those in 6-month-old rats, which was consistent with the neuroendocrine theory of human aging (Cutler and Hodes, 1983; Dai et al., 2009; Gaffey et al., 2016). That is, the hypothalamic-pituitary-adrenal axis (HPA), sympathetic-adrenal medulla axis (SAM), and (sympathetic) renin-angiotensin-aldosterone system are activated during aging, accompanied by the improvement of hormonal and endocrine functions. The serum MDA level of 18-month-old rats was significantly higher than that of 6-month-old rats, and the SOD activity level was significantly lower than that of 6-month-old rats, which was consistent with the free radical damage theory (Pan et al., 2002). The activities of LDH and CK in the serum of 18-month-old rats were significantly higher than those in the control group, but SDH was the opposite, which was consistent with fatigue caused by abnormal energy metabolism (Alexander et al., 2010; Zhao et al., 2015). The serum BNP level of 18-month-old rats was significantly higher than that of 6-month-old rats, indicating that aging is also accompanied by the decline of cardiac function. The results showed that the changes of physiological and biochemical indexes in 18-month-old rats reflected the aging phenomenon of natural aging animal model. GFR can effectively improve the abnormal indexes related to aging in 18-month-old rats, indicating that GFR has a positive effect on delaying aging.

With aging, the structure and function of the nervous system change, and there is an imbalance between transmitters, which causes aging changes in the function of the nervous system. In this study, the results of targeted metabolomics analysis of neurotransmitters in brain tissues showed that 9 neurotransmitters, related metabolites and their correlation ratios in brain tissues of 18-month-old rats were significantly different from those of 6-month-old rats. Among them, glutamic acid (Zahr et al., 2013; Huang et al., 2017), 5-hydroxytryptamine (Carfagna et al., 1985; Gottfries, 1985), gamma-aminobutyric acid (Cuypers et al., 2018) and dopamine (Simpkins et al., 1977) were basically consistent with literature reports (*p* < 0.05). NE is involved in regulating cardiovascular activity, body temperature, emotional activity and other physiological functions, and is also related to maintaining the awake state of the cerebral cortex. A large amount of NE act on α1-adrenergic receptors in the PVN and stimulate the hypothalamus to release more CRH, resulting in increased activity of the adrenocorticotropic system and enhanced peripheral sympathetic nerve activity (Dunn et al., 2004). GABA, formed by the decarboxylation of Glu in response to GAD, is the major inhibitory neurotransmitter in the central nervous system and has a sedative effect (Jakobs et al., 1993; Wong et al., 2003)., whereas Glu is the major excitatory neurotransmitter (Parsa et al., 2017; Weigend et al., 2019). Studies indicate that the Glu/GABA ratio can be utilized to evaluate the excitability or inhibitory state of the nervous system (Volgin et al., 2014; Shao et al., 2020). Histamine can regulate physiological functions, including the sleep-wake cycle, water and food intake, movement, neuroendocrine regulation, attention, learning, and memory by increasing neuronal excitability (Selbach et al., 1997) and promoting NMDA glutamatergic receptor-mediated responses (Bekkers, 1993). 5-HT can inhibit the function of NE neurons (Szabo and Blier, 2002), and the imbalance of the two may reduce the firing activity of NE neurons. After the intervention of GFR in 18-month-old rats, NE level decreased significantly, 5-HT/NE ratio, GABA, HA increased to different degrees. Pyruvate and α-ketoglutarate in alanine, aspartate and glutamate metabolism, and arginine and proline metabolism pathways are involved in the production of γ-aminobutyric acid. Urocanic acid and α-ketoglutaric acid in histidine metabolism are involved in the production of histamine. The tyrosine metabolic pathway affected the synthesis of NE. In previous studies, ginsenoside Rb1 and Re can reduce NE levels in the cortex and hypothalamus of stressed mice (Xu et al., 2009; Lee et al., 2012). Rb1 may inhibit presynaptic GABA_B_R1-enhanced GABA release and inhibitory transmission mediated by GABA_A_ receptors (Liu Y. et al., 2019). In conclusion, FGR can moderately suppress central nervous excitation by modulating the levels of aging central neurotransmitters such as NE, GABA, HA, and the Glu/GABA ratio. This regulation facilitates physiological functions including sedation and enhancement of learning and memory.

Cardiovascular aging is an important hub connecting aging and aging-related diseases (North and Sinclair, 2012), and is associated with atherosclerosis, hypertension, myocardial infarction and other cardiovascular diseases. B-type natriuretic peptide is a member of the natriuretic peptide system (NPs), which is synthesized by cardiomyocytes. It is a natural hormone with biological activities such as vasodilation, natriuresis, lowering blood pressure, inhibiting renin and aldosterone secretion, reducing sympathetic nervous system (SNS) tension, anti-myocardial fibrosis, and anti-myocardial hypertrophy (Volpe et al., 2016). It is also a gold index for non-traumatic evaluation of cardiac function (Hirata et al., 2001; Tsutamoto et al., 2001). AngⅡ is the most important active substance in the renin-angiotensin system (RAAS) and has many biological activities. It is closely related to ventricular hypertrophy and remodeling, heart failure, myocardial fibrosis, and vascular wall hypertrophy after vascular injury (Varo et al., 1999; Serneri et al., 2001; Yu and Liang, 2002). When the sympathetic nervous system is excited, the adrenal medulla secretion increases. At this time, NE in the blood is mainly from the sympathetic postganglionic fibers, and epinephrine E is mainly from the adrenal medulla. They act on adrenergic receptors, such as those expressed in the heart, triggering a positive inotropic response, stimulating myocardial contractility, and maintaining a compensatory mechanism of cardiac output (Lucia et al., 2018). Continuous activation of RAAS and SNS will eventually become harmful by aggravating myocardial cell damage, promoting ventricular remodeling, and then worsening cardiac function and the progression and deterioration of heart failure (Kemp and Conte, 2012). 20S-protopanaxdiol saponins derived from the leaves of P.quinquefolius (PQDS) have protective effects on acute myocardial ischemia. The mechanism may be related to the inhibition of SAM over-excitation, the reduction of catecholamine (CA) secretion and the inhibition of RAS activation, the reduction of AngⅡ production, and the break of the vicious cycle caused by mutual promotion of CA and RAS (Lu et al., 2001). The results of this study showed that the above indicators were significantly improved after the intervention of GFR in 18-month-old rats, which may be due to the inhibition of RAAS and SAM by central neurotransmitters, which also proved that GFR could regulate the cardiovascular function of aged rats through nervous and endocrine systems.

The body needs enough energy to sustain basic life activities. With increasing of age, the integrity of mitochondria and bioenergy efficiency decline, and the decrease of ATP production and insufficient energy supply lead to the decline of the body’s metabolic capacity, resulting in a series of aging changes, such as the change of enzyme activity (Srivastava, 2017). Succinate dehydrogenase (SDH) is a rate-limiting enzyme that regulates the glycolytic pathway and tricarboxylic acid cycle (Tan et al., 2014) and is necessary for catalyzing ATP synthesis, mainly distributed in the inner mitochondrial membrane. Changes in SDH activity are related to the number and damage of mitochondria, it is often considered a mitochondrial marker enzyme (Huang et al., 2005). The upregulation of SDH activity marks the acceleration of TCA and the increase in ATP. Lactate dehydrogenase (LDH) is an important enzyme in the anaerobic metabolism of glycogen. Changes in LDH activity can reflect the glycolytic capacity of muscle under anaerobic conditions (Kanda et al., 2014). Therefore, it is considered as a marker of anaerobic metabolic enzymes (Koukourakis et al., 2016). Creatine generates phosphocreatine (CP) under the action of creatine kinase (CK) to participate in ATP synthesis and form the cycle. The phosphoric acid system does not require the participation of oxygen and can provide energy for the body quickly and directly to maintain various life activities (Mendes and Tirapegui, 2002). However, ATP and CP have very small reserves in the body, which can only sustain the maximum intensity muscle contraction for a few seconds, so normal life activities cannot be maintained by the phosphoric acid system alone (Karlsson and Saltin, 1970; Karlsson et al., 1972). In this study, the activities of CK and LDH in the serum of aged rats increased, which had the same change trend as that of qi deficiency rats (Lin et al., 2016), and the activity of SDH decreased, suggesting that muscle energy metabolism and anaerobic energy metabolism increased, while aerobic energy metabolism decreased in aged rats. In metabolomic studies, butanoate metabolism belongs to carbohydrate metabolism, in which pyruvate and α-ketoglutarate are involved. Compared with the control group, the contents of both in the model group were significantly lower, which was the same as the previous study (Shen et al., 2016). 20(S)-PPD can inhibit the increase of corticosterone, lactate, lactate dehydrogenase, and creatinine levels induced by weight-bearing swimming and can also reduce glucose levels, and it is inferred that 20(S)-PPD has anti-fatigue effects (Oh et al., 2015). 20(S) -ginsenoside Rg3 inhibited the decrease of NADH dehydrogenase, succinate dehydrogenase, and cytochrome C oxidase activities and improved mitochondrial respiratory function (Tian et al., 2006). After GFR treatment, enzyme activity and body metabolites are improved. All of the results suggest that GFR can improve energy metabolism and alleviate fatigue in aged rats.

Oxidative damage is considered a key cause of age-related diseases in mammals. In order to protect cells from oxidative damage, there are a variety of antioxidant components in cells, such as glutathione (GSH) and superoxide dismutase (SOD). SOD is a natural scavenger of superoxide anion free radicals, which can eliminate harmful free radicals generated in cell metabolism and plays an important role in maintaining the integrity of cell membrane structure (Yoon et al., 2000). The increase in MDA concentration is thought to be caused by free radicals through lipid peroxidation, which can cause lipid oxidation of cell membranes and an increase in plasma lipid proteins (Ben-Shachar et al., 1991). Glutathione and taurine play important roles in cellular antioxidation in animals (Chang et al., 2004; Wu et al., 2004; Sinha et al., 2007). In Cysteine and methionine metabolism, S-adenosylmethionine is a direct product of methionine catalyzed by methionine adenosyltransferase, which generates S-adenosylhomocysteine under the action of DNA methyltransferase. It helps in the synthesis of cysteine, the precursor of taurine and glutathione (Bin et al., 2017). Disorders in glycerophospholipid metabolism usually result in rapid production and accumulation of free fatty acids and lysophospholipids, which have proinflammatory effects and inflammatory cytokine release, leading to oxidative stress and inflammatory responses (Sagy-Bross et al., 2015). Spermidine can trigger the autophagy process (Yamashita et al., 2004; Pekar et al., 2021), and as an autophagy regulator, it may induce LC3B and Beclin1-mediated autophagy in cochlear hair cells (HC) due to ROS-mediated oxidative damage through arginine and proline metabolism (Miao et al., 2022). In the study of biochemical indicators, MDA levels increased, and SOD activity decreased in aged rats. In the metabolomic research, PC40:6|PC18:1_22:5, PC40:7|PC18:1_22:6, PC40:8|PC20:4 _20:4, LPC17:0, LPC20:2 and LPA18:1 were significantly increased. 20(S) -ginsenoside Rg3 could significantly reduce mitochondrial MDA content, increase SOD activity, and inhibit excessive Ca^2+^ intake in brain nerve cells of rats with cerebral ischemia (Tian et al., 2006). Low dose of ginsenoside Rg1 could increase the content of serum SOD, and the difference was significant (*p* < 0.01) (Kong, 2021). Ginseng saponin Rb3 decreases isozyme, malondialdehyde, lactate dehydrogenase, endothelial diastolic factor levels and increase glutathione peroxidase and superoxide dismutase (SOD) levels in a dose-dependent (Wu and Jia, 2016). After receiving GFR, all indexes were significantly retracted. These results indicate that GFR decoction has good antioxidant activity, which is helpful in alleviating the harm of oxidative damage to the body and maintaining the body’s REDOX balance.

## Conclusion

Based on the combination of pharmacodynamic and metabolomics approaches, we identified biomarkers and altered metabolic pathways in aging model rats regulated by GFR. GFR modulates neurotransmitter equilibrium through alanine, aspartate and glutamate metabolism, arginine and proline metabolism, histidine metabolism, and tyrosine metabolism. GFR inhibits RAAS and SNS by increasing the level of central inhibitory neurotransmitters to achieve cardiovascular protection. GFR can improve energy metabolism and relieve fatigue by regulating SDH, LDH, CK activity, and butanoate metabolism. GFR can improve the antioxidant effect of the body by increasing SOD activity and regulating glycerophospholipid metabolism, arginine and proline metabolism and cysteine and methionine metabolism. These results suggest that GFR can effectively improve aging-related metabolic activities such as neuroendocrine, cardiovascular, energy metabolism, and oxidative stress, and has a certain role in delaying aging.

## Data Availability

The original contributions presented in the study are included in the article/Supplementary Material, further inquiries can be directed to the corresponding author.
