# Supplementary material for: Combined non-targeted and targeted metabolomics reveals the mechanism of delaying aging of Ginseng fibrous root
Source: Front Pharmacol. 2024 Jul 24;15:1368776. doi: 10.3389/fphar.2024.1368776 (PMC11303238; doi:10.3389/fphar.2024.1368776)
Supplement: Supplementary file 1 [file DataSheet1.docx]

Supplementary Material

**Supplementary Figure**

**Supplementary Figure S1**


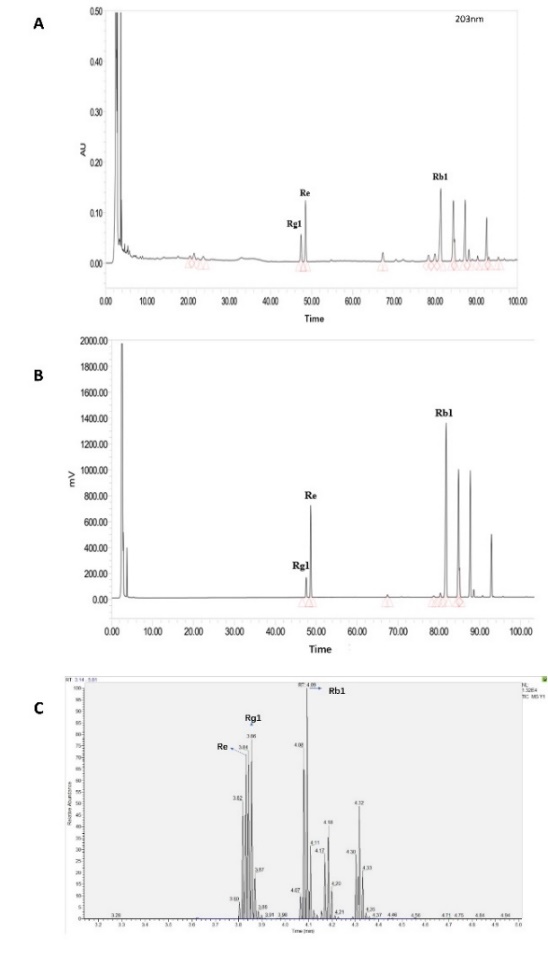


Supplementary Figure S1 Ginsenosides analysis of ginseng fibrous root decoction. (A)The UV signal showing the presence of ginsenosides. (B)The ELSD signal showing the presence of ginsenosides. (C)Total ion current chromatogram of typical ginseng fibrous root sample.

## Supplementary Table

**Supplementary Table S1.** Statistical analysis results of identified metabolite changes in serum

| No. | Adduct | Metabolite name | *M/Z* | RT/min | HMDB | KEGG | M vs.C | GFR vs. M | Pathway |
| --- | --- | --- | --- | --- | --- | --- | --- | --- | --- |
| 1 | [M-H]- | Alanine | 88.0406 | 0.73 | HMDB0000161 | C00041 | ↓^**^ | ↑^##^ | Amino acid metabolism |
| 2 | [M+FA-H]- | PC 40:6\|PC 18:1_22:5 | 878.5959 | 11.91 | HMDB0008088 | C00157 | ↑^*^ | ↓ | Lipid metabolism |
| 3 | [M+FA-H]- | PC 40:7\|PC 18:1_22:6 | 876.5800 | 11.64 | HMDB0008090 | C00157 | ↑^**^ | ↓ | Lipid metabolism |
| 4 | [M+FA-H]- | PC 40:8\|PC 20:4_20:4 | 874.5646 | 11.23 | HMDB0008443 | C00157 | ↑^*^ | ↓ | Lipid metabolism |
| 5 | [M+H] + | Threonine | 120.0656 | 0.66 | HMDB0000167 | C00188 | ↑^*^ | ↓^#^ | Amino acid metabolism |
| 6 | [M-H]- | Taurine | 124.0076 | 1.01 | HMDB0000251 | C00245 | ↓^**^ | - | Amino acid metabolism |
| 7 | [M-H]- | Quinic acid | 191.0565 | 0.76 | HMDB0003072 | C00296 | ↑^**^ | - | Amino acid metabolism |
| 8 | [M-H]- | Creatine | 130.0625 | 0.73 | HMDB0000064 | C00300 | ↓^**^ | ↑^##^ | Amino acid metabolism |
| 9 | [M-H]- | Isocitric acid | 191.0200 | 0.91 | HMDB0000193 | C00311 | ↑^*^ | ↓ | Carbohydrate metabolism |
| 10 | [M+H] + | Spermidine | 146.1652 | 0.51 | HMDB0001257 | C00315 | ↑^*^ | ↓ | Amino acid metabolism |
| 11 | [M-H]- | LPA 18:1 | 435.2528 | 9.93 | HMDB0007855 | C00416 | ↑^*^ | ↓ | Lipid metabolism |
| 12 | [M+H] + | Urocanic acid | 139.0503 | 1.17 | HMDB0000301 | C00785 | ↓^*^ | ↑^##^ | Histidine metabolism |
| 13 | [M+H] + | 4-Guanidinobutyric acid | 146.0927 | 1.04 | HMDB0003464 | C01035 | ↑^**^ | - | Arginine and proline metabolism |
| 14 | [M-H]- | Allantoin | 157.0369 | 0.70 | HMDB0000462 | C01551 | ↓^*^ | - | - |
| 15 | [M-H]- | Xanthosine | 283.0684 | 4.11 | HMDB0000299 | C01762 | ↑^*^ | - | Purine metabolism |
| 16 | [M-H]- | 2-Coumaric acid | 163.0406 | 5.52 | HMDB0002641 | C01772 | ↑^*^ | - | Phenylalanine metabolism |
| 17 | [M-H]- | Indolelactic acid | 204.0666 | 6.27 | HMDB0000671 | C02043 | ↓^*^ | ↑ | - |
| 18 | [M-H]- | Pimelic acid | 159.0666 | 5.85 | HMDB0000857 | C02656 | ↓^**^ | ↑^##^ | Metabolism of cofactors and vitamins |
| 19 | [M-H]- | N-Acetylleucine | 172.0982 | 6.13 | HMDB0011756 | C02710 | ↑^*^ | ↓^##^ | - |
| 20 | [M+H] + | Octanoylcarnitine | 288.2174 | 6.78 | HMDB0000791 | C02838 | ↑^*^ | ↓ | - |
| 21 | [M-H]- | LPA 16:0 | 409.2363 | 9.80 | HMDB0007853 | C04036 | ↑^*^ | ↓ | - |
| 22 | [M+FA-H]- | LPC 14:0 | 512.3004 | 8.77 | HMDB0010379 | C04230 | ↓^*^ | ↑ | Glycerophospholipid metabolism |
| 23 | [M+FA-H]- | LPC 17:0 | 554.3490 | 9.30 | HMDB0012108 | C04230 | ↑^*^ | ↓ | Glycerophospholipid metabolism |
| 24 | [M+FA-H]- | LPC 18:2 | 564.3335 | 8.99 | HMDB0010386 | C04230 | ↓^*^ | - | Glycerophospholipid metabolism |
| 25 | [M+FA-H]- | LPC 18:3 | 562.3172 | 8.82 | HMDB0010387 | C04230 | ↓^**^ | ↑ | Glycerophospholipid metabolism |
| 26 | [M+FA-H]- | LPC 20:2 | 592.3618 | 9.31 | HMDB0010392 | C04230 | ↑^*^ | ↓ | Glycerophospholipid metabolism |
| 27 | [M+FA-H]- | LPC 20:5 | 586.3160 | 8.80 | HMDB0010397 | C04230 | ↓^**^ | - | Glycerophospholipid metabolism |
| 28 | [M+H] + | 5-Hydroxy-3-indoleacetic acid | 192.0659 | 5.43 | HMDB0000763 | C05635 | ↓^*^ | ↑ | Tryptophan metabolism |
| 29 | [M-H]- | Panthenol | 204.1246 | 4.85 | HMDB0004231 | C05944 | ↓^*^ | ↑^#^ | Metabolism of cofactors and vitamins |
| 30 | [M+H] + | Muramic acid | 252.1079 | 0.70 | HMDB0003254 | C06470 | ↑^**^ | - | - |
| 31 | [M-H]- | Suberic acid | 173.0824 | 6.38 | HMDB0000893 | C08278 | ↓^*^ | - | - |
| 32 | [M-H]- | Skatole | 130.0663 | 6.72 | HMDB0000466 | C08313 | ↑^*^ | ↓^##^ | Tryptophan metabolism |
| 33 | [M-H]- | 11,12-Epoxyeicosatrienoic acid | 319.2288 | 8.75 | HMDB0004673 | C14770 | ↑^*^ | ↓ | Arachidonic acid metabolism |
| 34 | [M-H]- | Catechol | 109.0297 | 3.95 | HMDB0000957 | C00090 | ↑^*^ | - | Metabolic pathways |
| 35 | [M-H]- | Hexadecanedioic acid | 285.2072 | 8.58 | HMDB0000672 | C19615 | ↓^**^ | ↑ | Metabolic pathways |

Change trend compared with the control group: ↑ and ↓ represent the up-regulated and down-regulated expression of corresponding compounds in each group, respectively. “-”: No corresponding pathway. ^*^*P* < 0.05, ^**^*P* < 0.01, compared with the normal control group (C).; ^#^*P* < 0.05, ^##^*P* < 0.01, compared with the model control group (M). RT: retention time; C: normal control group; M: model control group; GFR: model administration group.

**Supplementary Table S2.** Statistical analysis results of identified metabolite changes in brain.

| No. | Adduct | Metabolite name | *M/Z* | RT/min | HMDB | KEGG | M vs. C | GFR vs. M | Pathway |
| --- | --- | --- | --- | --- | --- | --- | --- | --- | --- |
| 1 | [M-H]- | 2-Aminoadipic acid | 160.0616 | 0.79 | HMDB0000510 | C00956 | ↓^**^ | - | Lysine degradation |
| 2 | [M-H]- | Deoxyinosine | 251.0789 | 4.34 | HMDB0000071 | C05512 | ↓^**^ | ↑ | Purine metabolism |
| 3 | [M-H]- | 2-Oxoglutaric acid | 145.0144 | 1.15 | HMDB0000208 | C00026 | ↓^**^ | ↑ | Citrate cycle (TCA cycle) |
| 4 | [M-H]- | 3-Hydroxy-3-methylglutaric acid | 161.0459 | 2.61 | HMDB0000355 | C03761 | ↓^**^ | - | - |
| 5 | [M-H]- | 4-Hydroxyphenyllactic acid | 181.0506 | 5.46 | HMDB0000755 | C03672 | ↓^*^ | ↑^##^ | - |
| 6 | [M+H] + | 5-Hydroxy-3-indoleacetic acid | 192.0658 | 5.43 | HMDB0000763 | C05635 | ↓^**^ | - | Tryptophan metabolism |
| 7 | [M+H] + | 5-Methylthioadenosine | 298.0971 | 5.33 | HMDB0001173 | C00170 | ↓^**^ | - | Cysteine and methionine metabolism |
| 8 | [M-H]- | Abietic acid | 301.2173 | 9.38 | HMDB0247751 | C06087 | ↑^*^ | - | - |
| 9 | [M+H] + | Acetylcarnitine | 204.1231 | 1.08 | HMDB0000201 | C02571 | ↓^**^ | - | - |
| 10 | [M-H]- | Adenine | 134.0473 | 5.51 | HMDB0000034 | C00147 | ↓^*^ | ↑ | Purine metabolism |
| 11 | [M+H] + | Adenosine | 268.1039 | 3.26 | HMDB0000050 | C00212 | ↓^**^ | - | Purine metabolism |
| 12 | [M+NH4] + | alpha-Tocopherol acetate | 490.4256 | 8.88 | HMDB0034227 | C13202 | ↑^**^ | - | - |
| 13 | [M-H]- | Arachidonic acid | 303.2333 | 9.59 | HMDB0001043 | C00219 | ↑^*^ | - | Lipid metabolism |
| 14 | [M-H]- | Azelaic acid | 187.0975 | 6.87 | HMDB0000784 | C08261 | ↓^*^ | ↑ | - |
| 15 | [M+H] + | Carnitine | 162.1127 | 0.67 | HMDB0000062 | C00318 | ↓^*^ | - | - |
| 16 | [M+H] + | Corticosterone | 347.2218 | 7.56 | HMDB0001547 | C02140 | ↓^**^ | ↑ | Steroid hormone biosynthesis |
| 17 | [M-H]- | Cytidine | 242.0786 | 1.09 | HMDB0000089 | C00475 | ↓^*^ | - | Pyrimidine metabolism |
| 18 | [M+H] + | Cytosine | 112.0505 | 1.22 | HMDB0000630 | C00380 | ↓^*^ | - | Pyrimidine metabolism |
| 19 | [MH]- | Decanoic acid | 171.1394 | 7.42 | HMDB0000511 | C01571 | ↑^*^ | - | Fatty acid biosynthesis |
| 20 | [M-H]- | Deoxyuridine | 227.0674 | 3.40 | HMDB0000012 | C00526 | ↓^**^ | ↑ | Pyrimidine metabolism |
| 21 | [M+H] + | Diphenylamine | 170.0964 | 7.92 | HMDB0032562 | C11016 | ↑^*^ | - | - |
| 22 | [M-H]- | Docosahexanoic acid | 327.2334 | 9.56 | HMDB0002183 | C06429 | ↑^*^ | - | Biosynthesis of unsaturated fatty acids |
| 23 | [M-H]- | Eicosenoic acid | 309.2800 | 10.42 | HMDB0002231 | C16526 | ↑^*^ | - | Biosynthesis of unsaturated fatty acids |
| 24 | [M+H] + | Epigallocatechin | 307.0826 | 2.18 | HMDB0001871 | C09727 | ↓^**^ | - | - |
| 25 | [M-H]- | Nonadecanoic acid | 297.2805 | 10.62 | HMDB0000772 | C16535 | ↑^*^ | - | - |
| 26 | [M-H]- | gamma-Glutamylglutamine | 274.1041 | 0.79 | HMDB0011738 | C05283 | ↓^*^ | - | - |
| 27 | [M-H]- | Glutaric acid | 131.0353 | 3.84 | HMDB0000661 | C00489 | ↓^*^ | - | Amino acid metabolism |
| 28 | [M+H] + | Glutathione (oxidized) | 613.1600 | 2.18 | HMDB0003337 | C00127 | ↓^**^ | - | Glutathione metabolism |
| 29 | [M-H]- | Guanosine | 282.0841 | 4.04 | HMDB0000133 | C00387 | ↓^**^ | - | Purine metabolism |
| 30 | [M-H]- | Guanosine diphosphate mannose | 604.0728 | 1.01 | HMDB0001163 | C00096 | ↓^**^ | - | Amino acid metabolism |
| 31 | [M-H]- | GDP-L-fucose | 588.0753 | 1.3 | HMDB0001095 | C00325 | ↓^**^ | - | Carbohydrate metabolism |
| 32 | [M-H]- | Guanosine 5-monophosphate | 362.0515 | 1.35 | HMDB0001397 | C00144 | ↓^**^ | - | Purine metabolism |
| 33 | [M+H] + | Inosine | 269.0882 | 3.48 | HMDB0000195 | C00294 | ↓^*^ | - | Purine metabolism |
| 34 | [M-H]- | Inosine-5-monophosphate | 347.0400 | 1.32 | HMDB0000175 | C00130 | ↓^**^ | - | Purine metabolism |
| 35 | [M-H]- | Lauric acid | 199.1705 | 7.84 | HMDB0000638 | C02679 | ↑^**^ | - | Fatty acid biosynthesis |
| 36 | [M+H] **+** | Levodopa | 198.0737 | 4.46 | HMDB0000181 | C00355 | ↓^**^ | - | Tyrosine metabolism |
| 37 | [M-H]- | Lignoceric acid | 367.3581 | 12.61 | HMDB0002003 | C08320 | ↑^*^ | - | Biosynthesis of unsaturated fatty acids |
| 38 | [M-H]- | Linoleic acid | 279.2332 | 9.63 | HMDB0000673 | C01595 | ↑^*^ | - | Lipid metabolism |
| 39 | [M-H]- | Methyl Heptadecanoic acid | 283.2641 | 8.70 | HMDB0031066 | C20356 | ↑^*^ | - | - |
| 40 | [M-H]- | Myristic acid | 227.2014 | 8.20 | HMDB0000806 | C06424 | ↑^**^ | - | - |
| 41 | [M+H] + | N-Acetylaspartic acid | 176.0557 | 1.16 | HMDB0000812 | C01042 | ↓^**^ | - | Alanine, aspartate and glutamate metabolism |
| 42 | [M-H]- | N-Acetylaspartylglutamic acid | 303.0832 | 2.26 | HMDB0001067 | C12270 | ↓^**^ | - | Alanine, aspartate and glutamate metabolism |
| 43 | [M-H]- | N-Acetylglutamic acid | 188.0564 | 1.81 | HMDB0001138 | C00624 | ↓^**^ | - | Arginine biosynthesis |
| 44 | [M-H]- | Nervonic acid | 365.3430 | 11.75 | HMDB0002368 | C08323 | ↑^*^ | ↓^##^ | Biosynthesis of unsaturated fatty acids |
| 45 | [M+H] + | Niacinamide | 123.0552 | 1.33 | HMDB0001406 | C00153 | ↓^**^ | - | Nicotinate and nicotinamide metabolism |
| 46 | [M-H]- | Oleic acid | 281.2490 | 9.92 | HMDB0000207 | C00712 | ↑^*^ | - | Lipid metabolism |
| 47 | [M+H] + | Ophthalmic acid | 290.1339 | 1.47 | HMDB0005765 | C21016 | ↓^**^ | - | Cysteine and methionine metabolism |
| 48 | [M-H]- | Palmitic acid | 255.2328 | 9.82 | HMDB0000220 | C00249 | ↑^*^ | - | Lipid metabolism |
| 49 | [M-H]- | Pseudouridine | 243.0625 | 1.14 | HMDB0000767 | C02067 | ↓^**^ | ↑ | Pyrimidine metabolism |
| 50 | [M-H]- | Pyruvic acid | 87.0088 | 0.67 | HMDB0000243 | C00022 | ↓^*^ | ↑ | Citrate cycle (TCA cycle) |
| 51 | [M-H]- | Flavin mononucleotide | 455.0968 | 5.95 | HMDB0001520 | C00061 | ↓^*^ | - | Riboflavin metabolism |
| 52 | [M-H]- | Ribose-5-phosphate/Ribulose-5-phosphate/Xylulose-5-phosphate | 229.0119 | 0.67 | HMDB0001548 | C03736 | ↓^*^ | - | - |
| 53 | [M+H] + | S-Adenosyl-homocysteine | 385.1277 | 2.74 | HMDB0000939 | C00021 | ↓^*^ | ↑^##^ | Cysteine and methionine metabolism |
| 54 | [M-H]- | Sebacic acid | 201.1137 | 7.22 | HMDB0000792 | C08277 | ↓^*^ | ↑ | - |
| 55 | [M-H]- | Sinapyl alcohol | 209.0794 | 6.87 | HMDB0013070 | C02325 | ↓^*^ | ↑ | - |
| 56 | [M+H] + | Spermidine | 146.1652 | 0.49 | HMDB0001257 | C00315 | ↑^**^ | ↓ | Amino acid metabolism |
| 57 | [M+H] + | Sphingosine | 300.2891 | 8.20 | HMDB0000252 | C00319 | ↑^*^ | - | Sphingolipid metabolism |
| 58 | [M+H] + | Stearamide | 284.2946 | 10.31 | HMDB0034146 | C13846 | ↑^*^ | - | - |
| 59 | [M+H] + | Targinine | 189.1346 | 0.66 | HMDB0029416 | C03884 | ↑^*^ | - | - |
| 60 | [M+H] + | Taurine | 126.0220 | 0.63 | HMDB0000251 | C00245 | ↓^**^ | - | Taurine and hypotaurine metabolism |
| 61 | [M]+ | Thiamine monophosphate | 345.0774 | 0.69 | HMDB0002666 | C01081 | ↓^**^ | - | Taurine and hypotaurine metabolism |
| 62 | [M-H]- | Threonic acid | 135.0302 | 0.73 | HMDB0000943 | C01620 | ↓^**^ | ↑ | Ascorbate and aldarate metabolism |
| 63 | [M-H]- | Threonine | 118.0512 | 0.67 | HMDB0000167 | C00188 | ↑^*^ | ↓ | Amino acid metabolism |
| 64 | [M+H] + | Thymine | 127.0502 | 4.44 | HMDB0000262 | C00178 | ↓^**^ | - | Pyrimidine metabolism |
| 65 | [M-H]- | UDP-D-glucose | 565.0479 | 0.78 | HMDB0000286 | C00029 | ↓^**^ | - | Carbohydrate metabolism |
| 66 | [M-H]- | Uridine diphospho-N-acetylglucosamine | 606.0740 | 0.81 | HMDB0000290 | C00043 | ↓^**^ | - | Amino sugar and nucleotide sugar metabolism |
| 67 | [M-H]- | Uridine 5-monophosphate | 323.0293 | 1.02 | HMDB0000288 | C00105 | ↓^**^ | - | Pyrimidine metabolism |
| 68 | [M+H] + | Uridine diphosphoacetylgalactosamine | 608.0875 | 0.79 | HMDB0000304 | C00203 | ↓^**^ | - | Amino sugar and nucleotide sugar metabolism |
| 69 | [M+H] + | Urocanic acid | 139.0501 | 1.17 | HMDB0000301 | C00785 | ↓^**^ | ↑^#^ | Histidine metabolism |

Change trend compared with the control group: ↑ and ↓ represent the up-regulated and down-regulated expression of corresponding compounds in each group, respectively. “-”: No corresponding pathway. ^*^*P* < 0.05, ^**^*P* < 0.01, compared with the normal control group (C).; ^#^*P* < 0.05, ^##^*P* < 0.01, compared with the model control group (M). RT: retention time; C: normal control group; M: model control group; GFR: model administration group.
